# Identifying the Edges of the Optic Cup and the Optic Disc in Glaucoma Patients by Segmentation

**DOI:** 10.3390/s23104668

**Published:** 2023-05-11

**Authors:** Srikanth Tadisetty, Ranjith Chodavarapu, Ruoming Jin, Robert J. Clements, Minzhong Yu

**Affiliations:** 1 Department of Computer Science, Kent State University, Kent, OH 44242, USA; stadiset@kent.edu (S.T.); rchodava@kent.edu (R.C.);; 2 Department of Biological Sciences, Kent State University, Kent, OH 44242, USA; rclement@kent.edu; 3 Department of Ophthalmology, University Hospitals, Case Western Reserve University, Cleveland, OH 44106, USA

**Keywords:** segmentation, edge, detection, eye, diseases, glaucoma

## Abstract

With recent advancements in artificial intelligence, fundus diseases can be classified automatically for early diagnosis, and this is an interest of many researchers. The study aims to detect the edges of the optic cup and the optic disc of fundus images taken from glaucoma patients, which has further applications in the analysis of the cup-to-disc ratio (CDR). We apply a modified U-Net model architecture on various fundus datasets and use segmentation metrics to evaluate the model. We apply edge detection and dilation to post-process the segmentation and better visualize the optic cup and optic disc. Our model results are based on ORIGA, RIM-ONE v3, REFUGE, and Drishti-GS datasets. Our results show that our methodology obtains promising segmentation efficiency for CDR analysis.

## 1. Introduction

Fundus images are routinely used to detect eye diseases. Ophthalmologists used to analyze these images via a non-automated process, and it is a heavy burden for any ophthalmologist to read and explain the fundus images during the diagnosis of the ocular diseases [1]. Glaucoma is one of the major ocular diseases that causes visual impairment [2]. According to the World Health Organization (WHO), it has affected millions of people globally, and the early detection of glaucoma can prevent vision loss. The optic nerve transfers signals from the retina to the brain, whereby ganglion cell axons converge at the optic disc and exit the eye to form the optic nerve. The optic disc has a cup-shaped structure at the center, called the optic cup, which has a different color than the optic disc. In individuals with glaucoma, the size of the optic cup increases due to the death of the ganglion cells caused by the increase in intraocular pressure (IOP) and/or the loss of blood flow to the optic nerve. Therefore, the cup-to-disc ratio (CDR) is a main index for the early diagnosis of glaucoma and for the quantitative evaluation of the severity of glaucoma. The normal CDR is less than 0.5. A CDR less than 0.4 without an abnormally small optic disc size indicates a normal optic disc. In this stage, glaucoma must be diagnosed by IOP or other methods. If the CDR is between 0.5 and 0.8, it is considered the moderate stage of glaucoma. If the CDR is higher than 0.8, it is considered the severe stage of glaucoma [3]. With recent advancements in artificial intelligence, fundus images with different diseases can be classified automatically for the early diagnosis of diseases. The most widely used method in image classification networks is the application of convolutional neural networks. Many previous studies have used various pre-trained network architectures for the classification of images and various other methods to obtain the edges of the optic cup and the optic disc in fundus images. In the current study, several datasets of glaucoma fundus images were segmented and compared using our proposed deep learning methodology. U-Net is particularly effective for biomedical image segmentation tasks, such as cell and tissue segmentation [4]. U-Net outperforms other CNN architectures, such as VGG and ResNet, in these applications affirming its potential utility for the current task [4]. 

In this paper, we propose a new method to visualize the contours of the optic cup and disk. We implemented a modified U-Net for segmenting the optic cup and optic disc of the glaucoma images, later applying edge detection and dilation using the Canny edge filter. Our model is evaluated on four publicly available datasets namely ORIGA, RIM-ONE v3, REFUGE, and Drishti-GS. Our approach achieves a good performance measured using popular image segmentation metrics (IOU and Dice) in detecting early-stage CDR. 

## 2. Related Work

Several studies have aimed to segment fundus images. Among them, Cheng et al. are the first to utilize a clustering-based approach for the segmentation of both the optic disc and optic cup [5]. Sarkar et al. proposed the threshold-based approach for the segmentation of both the optic disc and optic cup on the RIM-ONE dataset [6]. Sun et al. used a deep object detection network for the joint localization and segmentation of the optic cup and disc on the ORIGA dataset [7]. Thakur et al. used a level-set based approach to adaptively regularize Kernel-based intuitionistic Fuzzy C means (LARKIFCM) for optic cup and disc segmentation on RIM-ONE and Drishti-GS datasets [8]. Sevastopolsky et al. used a modified U-Net for disc and cup segmentation on RIM-ONE-V3 and DRISHTI-GS datasets [9]. Kim et al. used an FCN (fully connected network) on the RIGA dataset [10]. Yu et al. uses Modified U-net from ResNet-34 for segmentation on Messidor and RIGA datasets [11]. Al-Bande et al. used Fully conventional Dense-Net for disc and cup segmentation [12].

Some recent studies consider adopting the state-of-the-art deep vision architectures. Guo et al. segmented the optic cup and optic disc of glaucoma images using segmentation models, such as DeepCDR, Wavelet, and their proposed modified U-Net++ [13]. Fu et al. segmented the disc and cup in glaucoma using polar transformation and the deep learning architecture named M-net. That network solves the segmentation of the optic disc and the optic cup in a single-stage multi-layer input and is shown to perform better on the ORIGA and SCES datasets compared to other segmentation models, such as U-net, Superpixel, LRR, etc. [14]. Bajwa et al. used G1020, a large publicly available dataset with 1020 fundus images for glaucoma classification. They obtained an accuracy of approximately 80% using the Inception V3 architecture [15]. Anitha et al. classified and segmented the glaucoma images using a trained DenseNet-201 classifier and U-Net segmentation model. They show their models perform better than other deep learning models, such as VGG19, Inception, ResNet, etc., on ORIGA dataset [16]. Juneja et al. segmented the optic disc and cup using a modified version of the U-Net architecture and tested on the DRISHTI-GS dataset [17]. Pascal et al. developed a model that simultaneously learns the segmentation and classification and tested on REFUGE [18]. Jiang et al. used a region-based convolutional neural network for joint optic cup and optic disc segmentation, which was shown to outperform other methods on the ORIGA dataset [19]. Gu et al. proposed a context encoder network, which gathered high-level data and saved them as spatial data for segmentation and was shown to perform better on DRIVE datasets [20]. Liu et al. proposed a multi-layer edge attention network that utilizes the edge information in the encoding stage [21]. Bajwa et al. evaluated the disc localization on the ORIGA dataset, which resulted in a 2.7% relative improvement over the state-of-the-art results on the ORIGA dataset [22]. Xie et al. proposed a novel fully convolutional network called SU-Net, which combines with the Viterbi algorithm to jointly decode the segmentation boundary [23]. Gao et al. developed a Recurrent Fully Convolution Network (RFC-Net) for the automatic joint segmentation of the optic disc and the optic cup, which can capture more high-level information and subtle edge information [24]. Hervella et al. developed a simultaneous classification of glaucoma and segmentation of the optic disc and cup by taking advantage of both pixel-level and image-level labels during network training. Additionally, the segmentation results allowed the extraction of relevant biomarkers such as the cup-to-disc ratio. They have evaluated the model using REFUGE and DRISHTI-GS datasets [25]. Parkhi et al. utilized DeepLabv3 and ensemble models to perform the segmentation of the optic disc and cup [26]. Zhou et al. developed a one-stage network named EfficientNet and Attention-based Residual Depth-Wise Separable Convolution (EARDS) for joint OD and OC segmentation [27]. Wu et al. developed a transformer-based conditional U-Net framework and a new Spectrum-Space Transformer to model the interaction between noise and semantic features. This architectural improvement leads to a new diffusion-based medical image segmentation method called MedSegDiff-V2 [28]. Sun et al. used ResFPN-Net to learn the boundary features and the inner relation between OD and OC for automatic segmentation [29]. Xue et al. used hybrid level set modeling for disc segmentation [30]. Zaaboub et al. proposed a two-stage (OD localization and segmentation) approach to detect the contour of the OD [31]. Liu et al. proposed a novel unsupervised model based on adversarial learning to perform the optic disc and cup segmentation [32]. Xiong et al. proposed a weak label-based Bayesian U-Net exploiting Hough transform-based annotations to segment the optic disc in fundus images. To achieve this, they built a probabilistic graphical model and explored a Bayesian approach with the state-of-the-art U-Net framework [33]. Wang et al. extended the EfficientNet-based U-Net, named EE-U-Net, for OD and OC segmentation [34].

## 3. Materials and Methods

### 3.1. Dataset

In this study, we introduce a modified U-Net model to perform edge segmentation and dilation (boundary thickening) using various datasets with different image resolutions: ORIGA (2499 × 2048), RIM-ONE v3 (1300 × 1100), REFUGE (2124 × 2056), and Drishti-GS (2049 × 1751) (Table 1). Datasets consist of images and masks, which are binary images consisting of zero-valued RGB pixels as background and RGB values greater than or equal to [128, 128, 128] at each pixel index *i* for objects of interest, keeping in mind the presence of gray and white labels.

### 3.2. Architecture

**Figure 1:**
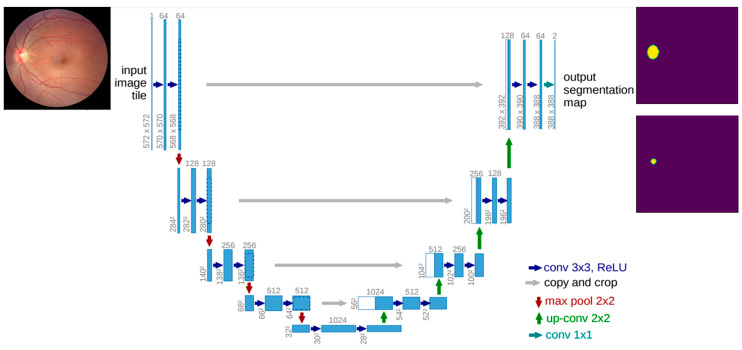
The network architecture of U-Net [3], including the input layer and convolution layer. Due to the size of the figure, the feature dimension is not scaled.

We use U-Net to extract features from the input fundus images and then convert the features into a high-level visual representation, which are processed for edge detection and dilation. The U-Net consists of an encoder and decoder. The encoder creates a compact representation of the input image (low dimension representation) to extract features via the convolution and pooling layers. The image is upsampled using the decoder, which reconstructs an image from the low dimensional representation. It too consists of the convolution block but has deconvolution layers to increase image dimensionality. The skip connections are the connections between the encoder and decoder that pass earlier features to the decoder. This helps the network capture the input an image’s low-level and high-level features. The skip connections are achieved by concatenating the encoder’s feature maps with the decoder’s corresponding feature maps at the same spatial resolution after the deconvolution [3]. After the initial convolution, the number of channels increases to 64. After the transposed convolution, the image is upsized from 28 × 28 × 1024 to 56 × 56 × 512 and concatenated with the contraction path skip connection image. The final layer is a 1 × 1 convolution to decrease the number of channels without affecting the image resolution (Figure 1). We limit the number of kernels to three for each layer convolution and implement a few pre-processing resizings to downsample the image and improve processing time.

### 3.3. Evaluation Criteria

Two widely used performance metrics were used for evaluating the segmentation results of the proposed model: (a) Dice Coefficient/F1 Score; (b) Jaccard Score/Intersection over union. The IoU represents the overlapping ratio between the segmentation results and ground truth mask. Both (a) and (b) are positively correlated.

(a) Dice Coefficient: Twice the area of the overlap divided by the total number of the pixels in both images (***A*** and ***B***).

(1) $$DC=\frac{2TP}{2TP+FP+FN}=\frac{2|A\cap B|}{2|A\cap B|+|B\backslash A|+|A\backslash B|}$$

where a true positive is represented by ***TP***, a false positive by ***FP***, and a false negative by ***FN*** [27].

(b) Jaccard Score: The area of overlap between the predicted image and the ground truth is divided by the area of union between the predicted image (***A***) and ground truth image (***B***).

(2) $$JAC=\frac{TP}{TP+FP+FN}=\frac{\left|A\cap B\right|}{\left|A|+|B|-|A\cap B\right|}$$

where a true positive is represented by ***TP***, a false positive is represented by ***FP***, and a false negative is represented by ***FN*** [27].

### 3.4. Edge Detection

**Figure 2:**
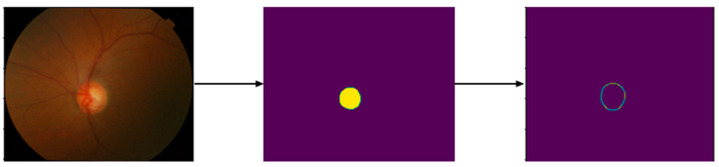
Sample result of Canny without dilation.

Canny is an edge detection operator that uses a multistage algorithm. It is composed of five steps: noise reduction, gradient calculation, non-maximum suppression, double threshold, and edge tracking by hysteresis. Noise is removed from the image by applying Gaussian blur via Gaussian kernels. Edges correspond to pixel intensity changes, which are detected by applying filters that highlight intensity changes in different directions (x,y). Non-max suppression is used to thin out the edges by going through all the points in the matrix ((i, j − 1), (i, j + 1), (i + 1,j), and (i − 1,j)) and suppressing (zeroing) non-max pixels. Double thresholding categorizes pixels into strong, weak, and other using a bounding threshold. The hysteresis will then transform weakly categorized pixels into strong ones [47] (Figure 2). Our method then applies a dilation on the resultant image to brighten the Canny generated edge. This entails convolving an image with a kernel that has a defined center. The max overlap pixel overlapped by the kernel is added to the image pixel at the kernel center position, thereby increasing the brightness [47].

## 4. Experimental Results

In this section, we present the various pre-processing steps and final output results from the different datasets. We carry out the experiments on an Intel California, USA manufactured Intel Xeon Platinum 8268 CPU @ 2.90 GHz running CentOS Stream 8 system with four Nvidia RTX 3090 GPUs having 24 GB of RAM. Each model is run for 300 epochs with a batch size of 4 using an Adam optimizer with a learning rate of 1 × 10^−4^.

### 4.1. Pre-Processing

**Figure 3:**
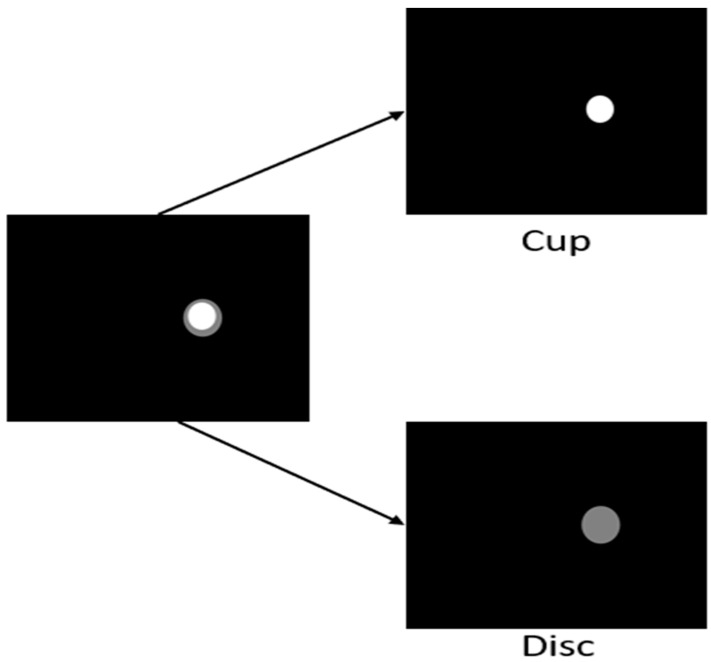
Pre-processing of the mask of the combined ORIGA and REFUGE datasets into separate cup and disc masks.

Datasets of various image dimensions are first resized to 256 × 256 for faster GPU processing. The ORIGA and REFUGE fundus images contained masks that have the cup and disc represented together. Since we are applying segmentation separately without having the cup segmentation hinder the disc or vice versa, the images are separated by changing the pixel values. White pixels are given the gray pixels’ values to form the disc images, and vice versa, to generate the cup images (Figure 3). This process was performed with the training, validation, and testing having an 80–10–10% data split, respectively. 

Data masks consist of RGB pixel values [0, 0, 0] for the background, and since we have white as cup and grey as disc, the model treats pixels greater than or equal to [128, 128, 128] as object labels (Figure 3).

To avoid overfitting on all datasets, training images were augmented with a random crop generated using the window width and height generated from a normal distribution, Gaussian blur, and random flip. All training, validation, and testing images were then normalized with pixel values between [−1, 1] after first resizing the image to 128 × 128 for model input.

### 4.2. Segmentation Results

**Figure 4:**
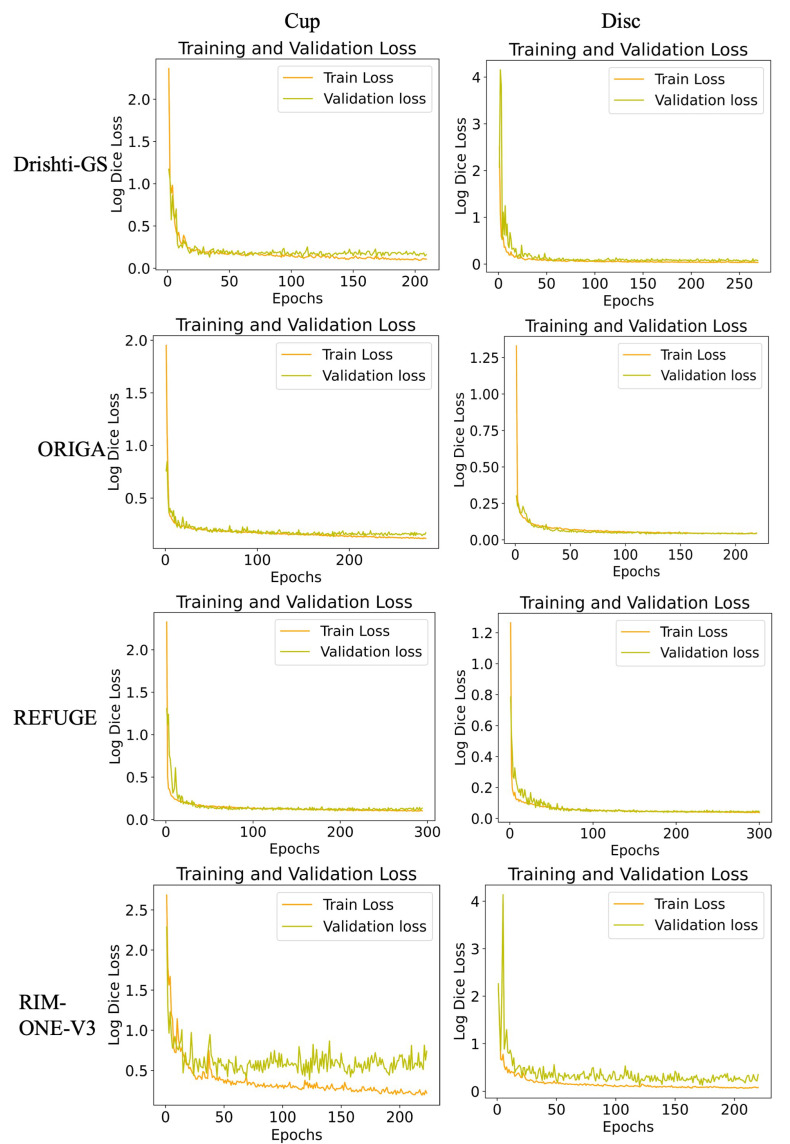
Loss function curves on various datasets (epoch vs. log Dice loss). Orange is the training loss and yellow is the validation loss.

**Figure 5:**
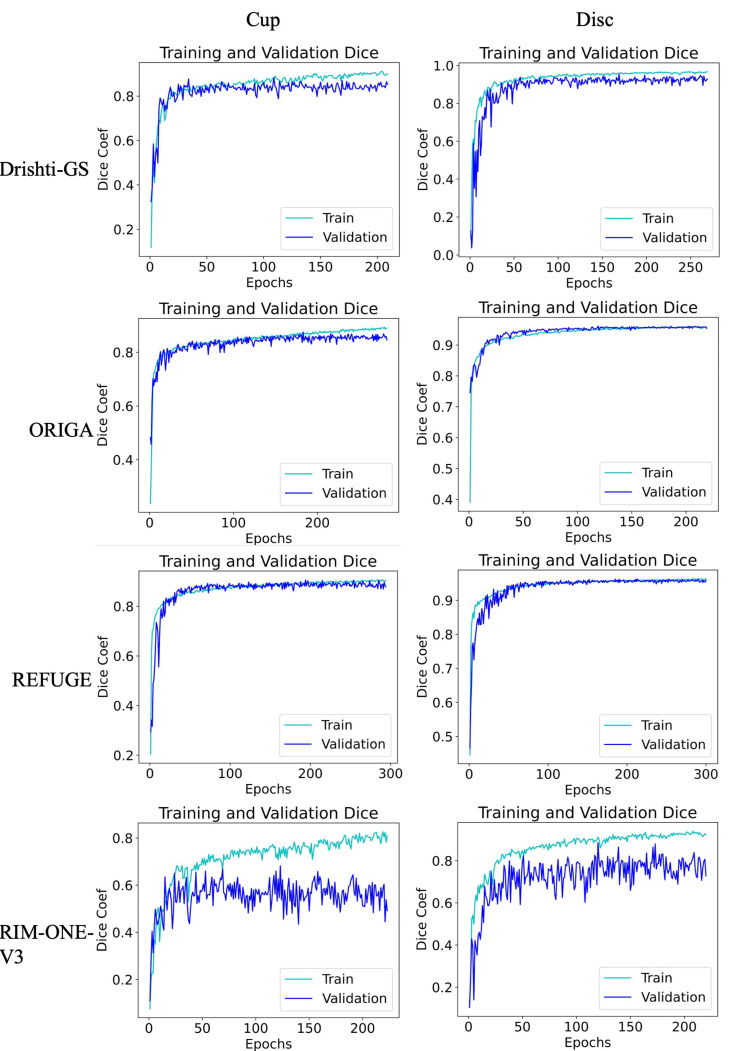
Accuracy (Jaccard Score) function curves on various datasets (epoch vs. Dice coefficient). Cyan is the train accuracy and blue is the validation accuracy.

We visualize the loss decrease over the training epochs and the accuracy (Jaccard Score) function curves for each dataset (Figure 4 and Figure 5, respectively). The loss steadily decreases except for the RIM-ONE-V3 dataset, which only consists of 74 images. This is the same result for the accuracy measure over 300 epochs.

Table 2 presents the Dice and IoU scores for each dataset using our model consisting of training parameters. Our model has 5,680,865 trainable parameters with 0 untrainable parameters and no frozen/dropped network nodes. On the Drishti-GS dataset, our approach achieves 0.058 and 0.117 for the best validation loss for the disc and cup, respectively. On the RIM-One-V3, it achieves 0.093 and 0.249 for the disc and cup, respectively. ORIGA achieves 0.037 and 0.137 for the disc and cup, respectively. Lastly, on the REFUGE dataset, validation loss achieves a minimal of 0.035 and 0.102 for disc and cup, respectively. 

With the Drishti-GS dataset, our approach achieves a 0.943 Dice and 0.893 IoU for OD segmentation. For OC segmentation, it achieves 0.889 Dice and 0.801 IoU. Using the RIM-One-V3 dataset, it obtains 0.910, 0.838 for Dice and IoU, respectively for OD segmentation, and it obtains 0.649, 0.77 for OC segmentation. With the ORIGA dataset, it achieves 0.962 and 0.928 for Dice and IoU, respectively for OD segmentation, and it obtains 0.871, 0.773 for OC segmentation. Lastly, with the REFUGE dataset, it acquires the scores of 0.965 and 0.933 (Dice and IoU respectively) for OD segmentation. This is followed by 0.902 and 0.824 for OC segmentation.

**Figure 6:**
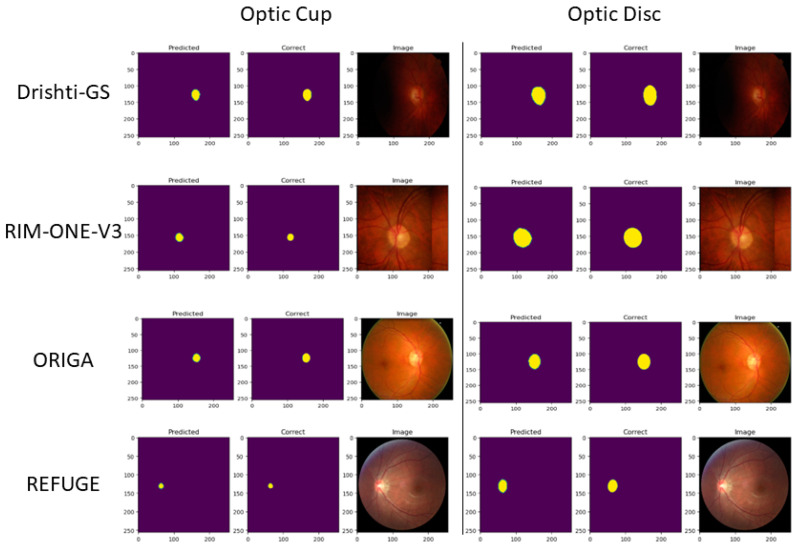
U-Net optic cup and disc segmentations without Canny and dilation for various datasets.

**Figure 7:**
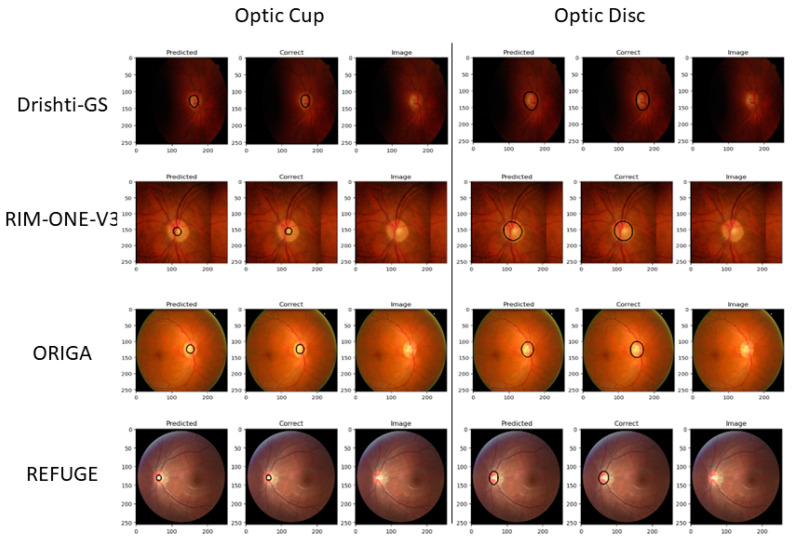
U-Net optic cup and disc segmentations with Canny and dilation for various datasets.

For visualizing the segmentation results, we randomly select images for all testing outputs from Drishti-GS, RIM-One-V3, ORIGA, and REFUGE. Refer to Figure 6 and Figure 7. Figure 6 shows the raw segmentation results without Canny and dilation applied. The first column is the prediction, followed by the ground truth, and the original image is to the right. The optic cup and disc segmentations have separate visualizations. Figure 7 shows the same results from Figure 6 with Canny and dilation applied to the resultant raw segmentation from our model output.

From Table 3, we achieved a ~94% Dice in OD segmentation and an 89% Dice in OC segmentation. In addition, we achieved a 0.89 Jaccard score in OD segmentation and a 0.8 Jaccard score in OC segmentation with the Drishti-GS dataset. Using the RIM-ONE-V3 dataset, we achieved a 91% Dice in OD segmentation and a 64% Dice in OC segmentation. Additionally, we achieved a 0.83 Jaccard for OD segmentation and a 0.77 Jaccard for OC segmentation. Our model achieved an approximate 97% Dice in OD segmentation and a 90% Dice in OC segmentation with the REFUGE dataset. The model also had a 0.93 Jaccard score OD segmentation and a 0.82 Jaccard score in OC segmentation with the REFUGE dataset. Lastly, using the ORIGA dataset, the model delivered a ~96% Dice and an ~87% Dice for OD and OC segmentation, respectively. Additionally, it delivered a 0.928 Jaccard and 0.773 Jaccard for OD and OC segmentation, respectively. 

## 5. Discussion

In this section, we start by comparing our U-Net model evaluation to those state-of-the-art approaches referenced in Table 3. While simple, our model performs on par with the presented models in the same datasets and performs slightly worse given Drishti-GS and RIM-ONE-V3 datasets due to the lack of image data. This is true for both the OD and OC segmentation results. On the other hand, our method performs slightly better than the other state-of-the-art models when run over the REFUGE and ORIGA datasets. Table 3 shows the related Dice and Jaccard metrics for both OD and OC segmentation, although most of the models do not run Jaccard for either OD or OC segmentation. Figure 6 and Figure 7 show results that are correlated with the model evaluation in Table 2 and Table 3. With the model performance being competitive, our representation of the edge detection and dilation deliver optimal results for CDR analysis for glaucoma. Concerning the results from the Drishti-GS dataset, FC-DenseNet [12] has a similar performance to our model for OD segmentation when we consider the Dice (0.949) and Jaccard (0.904) scores. For the OC segmentation, the multi-model [52] is also very similar in performance in terms of Dice (0.902) and Jaccard (0.822) scores. The results using the RIM-ONE-V3 dataset were comparable for OD segmentations. Both Drishit-GS and RIM-ONE-V3 have very small datasets. Our results for the ORIGA and REFUGE datasets are much higher with none of the existing models being comparable. We speculate that the reason for this is twofold: (1) both the REFUGE and ORIGA datasets have many images (650 and 1200, respectively) to scatter across training, validation and testing; (2) there is a clear color boundary that helps define the optic cup and optic disc, clearly helping the model better distinguish between them. The consistency in the sizes of masks in both datasets is indicative of this.

## 6. Conclusions

The work outlined herein displayed an end-to-end separative OD and OC segmentation approach. We first employ a modified U-Net encoder to find the features map and then a decoder to upsample the image back. The output is fed into an edge detector that gives a thin boundary around the edge. Dilation is next applied to thicken the edge boundary for a better visual representation. These results indicate that the boundary is accurate and can be subsequently used for analyzing CDR in instances of glaucoma. Based on these results, our model is as competent as the existing state-of-the-art models and performs better using both the ORIGA and REFUGE datasets even with a simple network architecture.

Further work should be directed towards having a CDR detector that optimizes (fixes) our segmentation results according to the correct ratio requirement. Although our model achieves robust results even with a slightly modified U-Net pipeline, it remains to be seen how changing the model and using various backbone training methods would impact performance. This includes the ensemble models that would average pixel classifications for the most accurate detection. It is also possible to first apply object detection to crop the data prior to segmentation. Note that the current studies are limited by the use of 2D data; the prospect of processing 3D fundus images would be an extension that provides spatial data for segmentation, potentially yielding better results because of the greater definition between disc and cup pixels. In summary, we have developed a simple novel architecture that performs as well as, and sometimes better than, existing methods that automate the processing of fundus images for assisting the analysis of CDR in instances of glaucoma.

## Figures and Tables

**Table 1:** Glaucoma segmentation datasets.

Dataset	Description	Reference
Drishti-GS [35,36]	It contains a total of 101 images. “http://cvit.iiit.ac.in/projects/mip/drishti-gs/mip-dataset2/Home.php (accessed on 19 March 2023)”	[11,37,38,39,40]
ORIGA	It has a total of 650 retinal images that are available publicly on Kaggle. “https://www.kaggle.com/datasets/arnavjain1/glaucoma-datasets?select=ORIGA (accessed on 19 March 2023)”	[38,40,41,42,43]
RIM-ONE-V3 [44]	RIM-ONE is a publicly available dataset of 74 colored fundus images. “http://medimrg.webs.ull.es/research/downloads/ (accessed on 19 March 2023)”	[37,38,40,45]
REFUGE [46]	It comprises 1200 colored retinal images with 400 images each for testing, validation, and training purposes. “https://www.kaggle.com/datasets/arnavjain1/glaucoma-datasets?select=REFUGE (accessed on 19 March 2023)”	[41]

**Table 2:** Dice and Jaccard evaluation metrics for various datasets.

Dataset	Optic Disc Segmentation		Optic Cup Segmentation	
	Dice/F1 Score	Jaccard Score/IoU	Dice/F1 Score	Jaccard Score/IoU
Drishti-GS	0.943	0.893	0.889	0.801
RIM-ONE-V3	0.910	0.838	0.649	0.770
ORIGA	0.962	0.928	0.871	0.773
REFUGE	0.965	0.933	0.902	0.824

**Table 3:** OD and OC segmentation results on Drishti-GS and REFUGE datasets.

Datasets	Methods	OD Segmentation		OC Segmentation	
		DC	JAC	DC	JAC
REFUGE	M-Net [12]	0.943	-	0.831	-
	M-Ada [25]	0.958	-	0.882	-
	EARDS [27]	0.954	0.914	0.887	0.801
	pOSAL [48]	0.946	-	0.875	-
	Multi-Model [49]	-	0.922	-	0.790
	CFEA [50]	0.941	-	0.862	-
	Two-Stage Mask R-CNN [51]	0.947	-	0.854	-
	***Ours***	***0.965***	***0.933***	***0.902***	***0.824***
ORIGA	Deep object detection Network [7]	0.845	-	0.845	-
	JointRCNN [19]	0.937	-	0.794	-
	SS-DCGAN [38]	0.901	-	-	-
	***Ours***	***0.962***	***0.928***	***0.871***	***0.773***
Drishti-GS	U-Net [3]	0.950	-	0.800	-
	[11]	0.973	0.949	0.887	0.804
	FC-DenseNet [12]	0.949	0.904	0.828	0.711
	M-Net [14]	0.959	-	0.866	-
	M-Ada [25]	0.971	-	0.910	-
	EARDS [27]	0.974	0.949	0.915	0.849
	ResFPN-Net [29]	0.976	-	0.896	-
	WRoIM [52]	0.960	-	0.890	-
	WGAN [53]	0.954	-	0.840	-
	pOSAL [48]	0.965	-	0.858	-
	GL-Net [54]	0.971	-	0.905	-
	Multi-Model [49]	0.960	0.924	0.902	0.822
	***Ours***	***0.943***	***0.893***	***0.889***	***0.801***
RIM-ONE -V3	Hybrid [8]	0.930	0.910	0.910	0.880
	Modified U-Net [9]	0.950	0.890	0.820	0.690
	ECSD [32]	0.860	0.760	0.800	0.680
	EE-U-Net [34]	0.950	0.880	0.860	0.760
	pOSAL [48]	0.860	-	0.787	-
	***Ours***	***0.910***	***0.830***	***0.640***	***0.770***

## Data Availability

Data sharing not applicable.

## References

[1] Abramoff M.D., Garvin M.K., Sonka M. (2010). Retinal imaging and image analysis. IEEE Rev. Biomed. Eng..

[2] Bourne R.R. (2006). Worldwide glaucoma through the looking glass. Br. J. Ophthalmol..

[3] Swetha M., Chitra Devi M., Jayashankari J.M.E., Veeralakshmi P. (2020). Automated Diagnosis of Glaucoma Using Cup to Disc Ratio. JETIR.

[4] Ronneberger O.F., Fischer P., Brox T. U-Net: Convolutional Networks for Biomedical Image Segmentation. Proceedings of the Medical Image Computing and Computer-Assisted Intervention–MICCAI 2015: 18th International Conference.

[5] Cheng J., Liu J., Xu Y., Yin F., Wong D.W.K., Tan N.-M., Tao D., Cheng C.-Y., Aung T., Wong T.Y. (2013). Superpixel Classification Based Optic Disc and Optic Cup Segmentation for Glaucoma Screening. IEEE Trans. Med. Imaging.

[6] Sarkar D.C., Das S., Bhattacharya I., Chakrabarti S., Reehal H., Lakshminarayanan V. (2017). Automated Glaucoma Detection of Medical Image Using Biogeography Based Optimization. Advances in Optical Science and Engineering.

[7] Sun X., Xu Y., Tan M., Fu H., Zhao W., You T., Liu J. Localizing Optic Disc and Cup for Glaucoma Screening via Deep Object Detection Networks. Proceedings of the Computational Pathology and Ophthalmic Medical Image Analysis: First International Workshop, COMPAY 2018, and 5th International Workshop, OMIA 2018.

[8] Thakur N., Juneja M. (2019). Optic disc and optic cup segmentation from retinal images using hybrid approach. Expert Syst. Appl..

[9] Sevastopolsky A. (2017). Optic disc and cup segmentation methods for glaucoma detection with modification of U-Net convolutional neural network. Pattern Recognit. Image Anal..

[10] Kim J., Tran L.Q., Chew E.Y., Antani S.K. Optic Disc and Cup Segmentation for Glaucoma Characterization Using Deep Learning. Proceedings of the 2019 IEEE 32nd International Symposium on Computer-Based Medical Systems (CBMS).

[11] Yu S., Xiao D., Frost S., Kanagasingam Y. (2019). Robust optic disc and cup segmentation with deep learning for glaucoma detection. Comput. Med. Imaging Graph. Off. J. Comput. Med. Imaging Soc..

[12] Al-Bander B., Williams B.M., Al-Nuaimy W., Al-Taee M.A., Pratt H., Zheng Y. (2018). Dense Fully Convolutional Segmentation of the Optic Disc and Cup in Colour Fundus for Glaucoma Diagnosis. Symmetry.

[13] Guo F., Li W., Tang J., Zou B., Fan Z. (2020). Automated glaucoma screening method based on image segmentation and feature extraction. Med. Biol. Eng. Comput..

[14] Fu H., Cheng J., Xu Y., Wong D.W.K., Liu J., Cao X. (2018). Joint Optic Disc and Cup Segmentation Based on Multi-Label Deep Network and Polar Transformation. IEEE Trans. Med. Imaging.

[15] Bajwa M.N.S., Singh G.A.P., Neumeier W., Malik M.I., Dengel A., Ahmed S. G1020: A Benchmark Retinal Fundus Image Dataset for Computer-Aided Glaucoma Detection. Proceedings of the 2020 International Joint Conference on Neural Networks (IJCNN).

[16] Sudhan M.B., Sinthuja M., Pravinth Raja S., Amutharaj J., Charlyn Pushpa Latha G., Sheeba Rachel S., Anitha T., Rajendran T., Waji Y.A. (2022). Segmentation and Classification of Glaucoma Using U-Net with Deep Learning Model. J. Healthc. Eng..

[17] Juneja M., Singh S., Agarwal N., Bali S., Gupta S., Thakur N., Jindal P. (2020). Automated detection of Glaucoma using deep learning convolution network (G-net). Multimed. Tools Appl..

[18] Pascal L., Perdomo O.J., Bost X., Huet B., Otalora S., Zuluaga M.A. (2022). Multi-task deep learning for glaucoma detection from color fundus images. Sci. Rep..

[19] Jiang Y., Duan L., Cheng J., Gu Z., Xia H., Fu H., Li C., Liu J. (2020). JointRCNN: A Region-Based Convolutional Neural Network for Optic Disc and Cup Segmentation. IEEE Trans. Biomed. Eng..

[20] Gu Z., Cheng J., Fu H., Zhou K., Hao H., Zhao Y., Zhang T., Gao S., Liu J. (2019). CE-Net: Context Encoder Network for 2D Medical Image Segmentation. IEEE Trans. Med. Imaging.

[21] Liu H., Feng Y., Xu H., Liang S., Liang H., Li S., Zhu J., Yang S., Li F. (2022). MEA-Net: Multilayer edge attention network for medical image segmentation. Sci. Rep..

[22] Bajwa M.N., Malik M.I., Siddiqui S.A., Dengel A., Shafait F., Neumeier W., Ahmed S. (2019). Two-stage framework for optic disc localization and glaucoma classification in retinal fundus images using deep learning. BMC Med. Inform. Decis. Mak..

[23] Xie Z., Ling T., Yang Y., Shu R., Liu B.J. (2020). Optic Disc and Cup Image Segmentation Utilizing Contour-Based Transformation and Sequence Labeling Networks. J. Med. Syst..

[24] Gao J., Jiang Y., Zhang H., Wang F. (2020). Joint disc and cup segmentation based on recurrent fully convolutional network. PLoS ONE.

[25] Hervella Á.S., Rouco J., Novo J., Ortega M. (2021). End-to-end multi-task learning for simultaneous optic disc and cup segmentation and glaucoma classification in eye fundus images. Appl. Soft Comput..

[26] Parkhi P., Hambarde B.H. (2023). Optical Cup and Disc Segmentation using Deep Learning Technique for Glaucoma Detection. Int. J. Next Gener. Comput..

[27] Zhou W., Ji J., Jiang Y., Wang J., Qi Q., Yi Y. (2023). EARDS: EfficientNet and attention-based residual depth-wise separable convolution for joint OD and OC segmentation. Front. Neurosci..

[28] Wu J., Fu R., Fang H., Zhang Y., Xu Y. (2023). MedSegDiff-V2: Diffusion based Medical Image Segmentation with Transformer. arXiv.

[29] Sun G., Zhang Z., Zhang J., Zhu M., Zhu X., Yang J., Li Y. (2021). Joint optic disc and cup segmentation based on multi-scale feature analysis and attention pyramid architecture for glaucoma screening. Neural Comput. Appl..

[30] Xue X., Wang L., Du W., Fujiwara Y., Peng Y. (2022). Multiple Preprocessing Hybrid Level Set Model for Optic Disc Segmentation in Fundus Images. Sensors.

[31] Zaaboub N., Sandid F., Douik A., Solaiman B. (2022). Optic disc detection and segmentation using saliency mask in retinal fundus images. Comput. Biol. Med..

[32] Liu B., Pan D., Shuai Z., Song H. (2022). ECSD-Net: A joint optic disc and cup segmentation and glaucoma classification network based on unsupervised domain adaptation. Comput. Methods Programs Biomed..

[33] Xiong H., Liu S., Sharan R.V., Coiera E., Berkovsky S. (2022). Weak label based Bayesian U-Net for optic disc segmentation in fundus images. Artif. Intell. Med..

[34] Wang J., Li X., Cheng Y. (2023). Towards an extended EfficientNet-based U-Net framework for joint optic disc and cup segmentation in the fundus image. Biomed. Signal Process. Control.

[35] Sivaswamy J., Krishnadas S.R., Joshi G.D., Jain M., Tabish A.U. Drishti-gs: Retinal image dataset for optic nerve head(onh) segmentation. Proceedings of the 2014 IEEE 11th International Symposium on Biomedical Imaging (ISBI).

[36] Sivaswamy J., Krishnadas S.R., Chakravarty A., Joshi G.D., Tabish A.U. (2015). A comprehensive retinal image dataset for the assessment of glaucoma from the optic nerve head analysis. JSM Biomed. Imaging Data Pap..

[37] Diaz-Pinto A., Morales S., Naranjo V., Köhler T., Mossi J.M., Navea A. (2019). CNNs for automatic glaucoma assessment using fundus images: An extensive validation. Biomed. Eng. Online.

[38] Diaz-Pinto A., Colomer A., Naranjo V., Morales S., Xu Y., Frangi A.F. (2019). Retinal Image Synthesis and Semi-Supervised Learning for Glaucoma Assessment. IEEE Trans. Med. Imaging.

[39] Zilly J., Buhmann J.M., Mahapatra D. (2017). Glaucoma detection using entropy sampling and ensemble learning for automatic optic cup and disc segmentation. Comput. Med. Imaging Graph. Off. J. Comput. Med. Imaging Soc..

[40] Phasuk S., Tantibundhit C., Poopresert P., Yaemsuk A., Suvannachart P., Itthipanichpong R., Chansangpetch S., Manassakorn A., Tantisevi V., Rojanapongpun P. Automated Glaucoma Screening from Retinal Fundus Image Using Deep Learning. Proceedings of the 2019 41st Annual International Conference of the IEEE Engineering in Medicine and Biology Society.

[41] Wang J., Yan Y., Xu Y., Zhao W., Min H., Tan M., Liu J. Conditional Adversarial Transfer for Glaucoma Diagnosis. Proceedings of the 2019 41st Annual International Conference of the IEEE Engineering in Medicine and Biology Society.

[42] Chen X., Xu Y., Wong D.W.K., Wong T.Y., Liu J. Glaucoma detection based on deep convolutional neural network. Proceedings of the 2015 37th Annual International Conference of the IEEE Engineering in Medicine and Biology Society.

[43] Li A., Cheng J., Wong D.W.K., Liu J. Integrating holistic and local deep features for glaucoma classification. Proceedings of the 2016 38th Annual International Conference of the IEEE Engineering in Medicine and Biology Society (EMBC).

[44] Fumero F., Sigut J.F., Alayón S., Gonzalez-Hernandez M., Rosa M.G. Interactive Tool and Database for Optic Disc and Cup Segmentation of Stereo and Monocular Retinal Fundus Images. Proceedings of the 23rd International Conference in Central Europe on Computer Graphics, Visualization and Computer Vision 2015 in Co-Operation with EUROGRAPHICS.

[45] Cerentini A., Welfer D., d’Ornellas M.C., Pereira Haygert C.J., Dotto G.N. (2017). Automatic Identification of Glaucoma Using Deep Learning Methods. MEDINFO 2017: Precision Healthcare Through Informatics: Proceedings of the 16th World Congress on Medical and Health Informatics.

[46] Orlando J.I., Fu H., Breda J.B., van Keer K., Bathula D.R., Diaz-Pinto A., Fang R., Heng P.-A., Kim J., Lee J. (2020). Refuge challenge: A unified framework for evaluating automated methods for glaucoma assessment from fundus photographs. Med. Image Anal..

[47] Ding L., Goshtasby A. (2001). On the Canny edge detector. Pattern Recognit..

[48] Wang S., Yu L., Yang X., Fu C.W., Heng P.A. (2019). Patch-Based Output Space Adversarial Learning for Joint Optic Disc and Cup Segmentation. IEEE Trans. Med. Imaging.

[49] Hervella Á.S., Ramos L., Rouco J., Novo J., Ortega M. Multi-Modal Self-Supervised Pre-Training for Joint Optic Disc and Cup Segmentation in Eye Fundus Images. Proceedings of the ICASSP 2020—2020 IEEE International Conference on Acoustics, Speech and Signal Processing (ICASSP).

[50] Liu P., Kong B., Li Z., Zhang S., Fang R. (2019). CFEA: Collaborative Feature Ensembling Adaptation for Domain Adaptation in Unsupervised Optic Disc and Cup Segmentation. Medical Image Computing and Computer Assisted Intervention—MICCAI 2019.

[51] Almubarak H., Bazi Y., Alajlan N. (2020). Two-Stage Mask-RCNN Approach for Detecting and Segmenting the Optic Nerve Head, Optic Disc, and Optic Cup in Fundus Images. Appl. Sci..

[52] Shah S., Kasukurthi N., Pande H. (2019). Dynamic region proposal networks for semantic segmentation in automated glaucoma screening. Proceedings of the 2019 IEEE 16th International Symposium on Biomedical Imaging (ISBI 2019).

[53] Kadambi S., Wang Z., Xing E. (2020). WGAN domain adaptation for the joint optic disc-and-cup segmentation in fundus images. Int. J. Comput. Assist. Radiol. Surg..

[54] Jiang Y., Tan N., Peng T. (2019). Optic Disc and Cup Segmentation Based on Deep Convolutional Generative Adversarial Networks. IEEE Access.

